# Lipids and transaminase elevations in ARV-experienced PLWH switching to a doravirine-based regimen from rilpivirine or other regimens

**DOI:** 10.1186/s12879-023-08191-2

**Published:** 2023-04-14

**Authors:** Paolo Maggi, Elena Delfina Ricci, Stefania Cicalini, Giovanni Francesco Pellicanò, Benedetto Maurizio Celesia, Francesca Vichi, Antonio Cascio, Eleonora Sarchi, Giancarlo Orofino, Nicola Squillace, Giordano Madeddu, Giuseppe Vittorio De Socio, Olivia Bargiacchi, Chiara Molteni, Addolorata Masiello, Annalisa Saracino, Barbara Menzaghi, Katia Falasca, Lucia Taramasso, Antonio Di Biagio, Paolo Bonfanti

**Affiliations:** 1 Infectious Diseases Unit, AORN Sant’Anna e San Sebastiano, Caserta, Italy; 2 Fondazione ASIA Onlus, Via Garibaldi, 13, Buccinasco, 20090 MI Italy; 3 National Institute for Infectious Diseases Lazzaro Spallanzani Institute for Hospitalization and Care Scientific, Roma, Lazio, Italy; 4 Unit of Infectious Diseases, Department of Human Pathology of the Adult and the Developmental Age ‘G. Barresi’, University of Messina, Messina, Italy; 5 Unit of Infectious Diseases, Garibaldi Hospital, Catania, Italy; 6 Unit of Infectious Diseases, USLCENTRO FIRENZE, Santa Maria Annunziata Hospital, Florence, Italy; 7 Unit of Infectious Diseases, Department of Health Promotion, Mother and Child Care, Internal Medicine and Medical Specialties, University of Palermo, Palermo, Italy; 8 Infectious Diseases Unit, S.Antonio e Biagio e Cesare Arrigo Hospital, Alessandria, Italy; 9 Division I of Infectious and Tropical Diseases, ASL Città di Torino, Torino, Italy; 10 Infectious Disease Unit, Fondazione IRCCS San Gerardo dei Tintori, Monza, Italy; 11 Unit of Infectious Diseases, Department of Medicine, Surgery and Pharmacy, University of Sassari, Sassari, Italy; 12 Unit of Infectious Diseases, Santa Maria Hospital, Perugia, Italy; 13 Unit of Infectious Diseases, Ospedale Maggiore della Carità, Novara, Italy; 14 Unit of Infectious Diseases, A. Manzoni Hospital, Lecco, Italy; 15 Department of Biomedical Sciences and Human Oncology, Clinic of Infectious Diseases, University of Bari Aldo Moro, Bari, Italy; 16 Unit of Infectious Diseases, ASST della Valle Olona – Busto Arsizio (VA), Busto Arsizio VA, Italy; 17 Clinic of Infectious Diseases, Department of Medicine and Science of Aging, G. D’Annunzio University, Chieti-Pescara, Chieti, Italy; 18 Infectious Diseases, San Martino Hospital Genoa, University of Genoa, Genoa, Italy; 19 Infectious Disease Unit, Fondazione IRCCS San Gerardo dei Tintori, Monza - University of Milano-Bicocca, Monza, Italy

**Keywords:** HIV infection, ART-experienced, Doravirine, Rilpivirine, Adverse events, Metabolic safety, Hepatic safety

## Abstract

**Background:**

Doravirine (DOR) is a newly approved antiretroviral belonging to the class of non-nucleoside reverse transcriptase inhibitors (NNRTI), well tolerated and leading to an improved lipid profile in antiretroviral experienced people living with HIV (PLWH). We aimed at evaluating if the lipid-lowering effect is linked to the drug class, using real-life data from the SCOLTA cohort.

**Methods:**

We compared the lipid profile modifications in experienced PLWH switching to a DOR-based regimen from rilpivirine or another NNRTI-based regimen or from an integrase strand transferase (INSTI)-based regimen. T0 and T1 were defined as the baseline and 6-month follow-up respectively. Data were collected at baseline and prospectively every six months and changes from baseline were compared using a multivariable linear model.

**Results:**

In 107 PLWH, enrolled in the SCOLTA DOR cohort, with undetectable HIV-RNA at baseline, 32.7% switched from RPV-based regimens (DOR1), 29.9% from other NNRTI-including regimens (DOR2) and 37.4% switched from INSTI-including regimens (DOR3). At T1, TC significantly decreased in DOR2 (-15 mg/dL) and DOR3 (-23 mg/dL), and significantly more in DOR3 than in DOR1 (-6 mg/dL) (p = 0.016). HDL-C declined in DOR2 (-2 mg/dL) whereas it increased in DOR1 (+ 3 mg/dL) (p = 0.042) and remained stable in DOR3. LDL-C significantly decreased from baseline in DOR2 (-12 mg/dL) and DOR3 (-22 mg/dL) and was different between DOR1 (-8 mg/dL) and DOR3 (p = 0.022). TC/HDL ratio showed a significant decline in the DOR3 group (-0.45), although similar to DOR1 (-0.23, p = 0.315) and DOR2 (-0.19, p = 0.254). Triglycerides did not noticeably change. ALT significantly decreased in PLWH with a baseline level > 40 UI/mL.

**Conclusions:**

PLWH on doravirine treatment showed different trends in blood lipids according to their previous regimen. In PLWH switching from RPV, minimal modifications were seen, whereas in those switching from other NNRTIs and from INSTI-including regimens, we observed an overall improvement in lipid profile, seemingly independent of the “statin effect” of TDF.

## Introduction

Doravirine (DOR) is a newly approved antiretroviral belonging to the class of non-nucleoside reverse transcriptase inhibitors (NNRTI). This agent seems well tolerated both in antiretroviral (ART)-naïve and ART-experienced people living with HIV (PLWH), leading to an improved lipid profile [1, 2].

DOR and rilpivirine (RPV), another second-generation NNRTI approved in 2011, are generally considered safe drugs, especially regarding cardiovascular impact [3–5].

As for hepatic safety, the NNRTIs were considered a class at risk for aspartate aminotransferase (AST) and/or alanine aminotransferase (ALT) elevation [6], with first-generation NNRTIs such as nevirapine and efavirenz largely related to hepatic toxicity [7]. On the contrary, second-generation drugs (etravirine, RPV, and DOR) seem generally safe for the liver, even in people co-infected with hepatitis viruses [6, 8–10]. Regarding DOR, until now serum aminotransferase elevations have been reported in 13% of PLWH on regimens containing this drug, but elevations above five times the upper limit of normal are uncommon, occurring in 1% or less of people [11]. DOR has not been linked to cases of acute hepatitis, acute liver failure, chronic hepatitis, or vanishing bile duct syndrome [11].

Whereas for RPV and other NNRTIs the bulk of evidence is substantial, as regards DOR both metabolic and hepatic safety were mainly analyzed in phase 3 studies [1, 2]. Thus, we used information from a real-life setting to provide new data on these issues. To evaluate if the lipid-lowering effect is linked to the drug class or DOR-specific, we compared PLWH switching to a DOR-based regimen from an RPV-including regimen, from one including an NNRTI other than RPV (thus including older NNRTIs, nowadays less frequently used in real-world scenarios), and from integrase strand transfer inhibitors (INSTI). Since information on the hepatic safety of DOR is scarce, we also analyzed the ALT trend during the first months of observation.

## Methods

The SCOLTA (Surveillance COhort Long Term Toxicity Antiretrovirals/antivirals) project is a multicenter observational study established in 2002 and following prospectively PLWH who start to take new antiretroviral drugs, to identify toxicities and adverse events (AEs) in a real-life setting [12]. The SCOLTA project uses an online pharmacovigilance program and involves 25 Italian Infectious Disease Centers (www.cisai.it). Briefly, both ART naïve and ART experienced PLWH aged 18 or more years, who start taking a newly marketed drug, are consecutively asked to give written informed consent and are then enrolled and included in the cohort for that drug. At baseline, clinical data collected include sex, age, ethnicity, weight, height, CDC stage, previous ART history, and information on comorbidities. Laboratory data include HIV-RNA, CD4 + T cell count, and biochemical data, which are prospectively collected in anonymous form in a central database. PLWH are followed up at a 6-month interval, and those who do not present to the physician in more than 6 months leave the study and are considered lost to follow-up. If individuals stop using the cohort drug, they leave the study. AEs are collected prospectively as soon as they are clinically observed.

The first participant was enrolled in the DOR cohort in February 2020 and the enrolment is still ongoing. The last individual included in the present analysis was enrolled in May 2022.

The original study protocol was approved on 18 September 2002, and a new protocol amendment was approved on 13 June 2013 by the coordinating center at Hospital “L. Sacco”-University of Milan, Milan (Italy) and thereafter by all participating centers. Written consent for study participation was obtained from all participants, and the study was conducted in accordance with the ethical standards laid down in the 1964 Declaration of Helsinki and its later amendments and by Italian national laws.

Data were described using mean and standard deviation (SD) for normally distributed continuous variables, median and interquartile range (IQR) for not normally distributed continuous variables, and frequency (%) for categorical and ordinal variables. Intragroup change from baseline was evaluated using the paired t-test. The intergroup changes from baseline were compared using a general linear multivariate model, where the age at study entry, the initial level of the variable, the current regimen, the concurrent use of lipid-lowering drugs, and TAF in the previous regimen were included as confounders.

## Results

We selected 107 experienced PLWH, enrolled in the SCOLTA DOR cohort, with undetectable HIV-RNA at baseline, and at least one visit after enrollment. PLWH with protease inhibitors (PI) in the previous regimen were excluded, based on the previous results which showed that DOR had a better lipid profile compared to PIs [1].

Thirty-five (32.7%) switched from RPV-based regimens (DOR1), 32 (29.9%) from other NNRTI-including regimens (DOR2) and 40 (37.4%) switched from INI-including regimens (DOR3). T0 and T1 were defined as the baseline and 6-month follow-up respectively.

The three groups did not significantly differ in terms of sex (female 34.3% vs. 28.1% vs. 22.5%, p = 0.52), ethnicity (Caucasian 94.3% vs. 93.0% vs. 87.5%, p = 0.50), and use of lipid-lowering drugs at baseline or during the first 6 months of observation (25.7% vs. 21.9% vs. 22.5%, p = 0.92). They differed in age (48.8 vs. 55.9 vs. 50.5 respectively, p = 0.03) and risk factor for HIV acquisition (past use of intravenous drugs 2.9% vs. 21.9% vs. 12.5% respectively, p = 0.02). Despite the higher prevalence of HCV positivity in DOR3 subjects (25.0% vs. 5.7% in DOR1 and 12.5% in DOR2, p = 0.06), abnormal ALT levels (> 40 UI/dL) were similar by group (Table).

Overall, in this study, 65.4% of PLWH were on lamivudine (3TC)/ tenofovir disoproxil fumarate (TDF)/DOR, 14.0% on emtricitabine (FTC)/ tenofovir alafenamide (TAF)/DOR and 20.6% on dual therapy with DOR/INSTI.

At baseline, mean high-density lipoprotein cholesterol (HDL-C) was higher in DOR2 than in DOR1 and DOR3, whereas total cholesterol (TC), TC/HDL-C ratio, low-density lipoprotein cholesterol (LDL-C), and triglycerides were similar or, at least, not significantly different (Table).

Changes from baseline were analyzed including the age at study entry, the initial level of the variable, the current regimen, the concurrent use of lipid-lowering drugs and TAF in the previous regimen, as confounders for the effect of the switch from RPV-based, other NNRTI-based and INSTI-based regimens. At T1, TC did not change in DOR1 (-6 mg/dL, 95% CI -18, +6), significantly decreased in DOR2 (-15 mg/dL, 95% CI -26, -5) and DOR3 (-23 mg/dL, 95% CI -32, -13), and significantly more in DOR3 than in DOR1 (p = 0.016), whereas the difference between DOR1 and DOR2 was not statistically significant (p = 0.235). HDL-C showed a more marked decline in DOR2 (-2 mg/dL, 95% CI -6, +2) whereas it increased in DOR1 (3 mg/dL, 95% CI -1, +8) (p = 0.042) and remained stable in DOR3 (0 mg/dL, 95% CI -3, +3). LDL-C significantly decreased from baseline in DOR2 (-12 mg/dL, 95% CI -22, -3) and DOR3 (-22 mg/dL, 95% CI -31, -14) and was different between DOR1 (-8 mg/dL, 95% CI -18, +3) and DOR3 (p = 0.022). TC/HDL ratio showed a marked decline in the DOR3 group (-0.45, 95% CI -0.75, -0.15), however similar to DOR1 (-0.23, 95% CI -0.61, +0.14) (p = 0.315) and DOR2 (-0.19, 95% CI -0.54, +0.16) (p = 0.254). Triglycerides did not noticeably change.

Then, we focused the analysis on 73 PLWH from non-TDF including regimens, comparing the lipid variations between 38 PLWH with TDF and 35 without TDF in the current regimen. Modifications were statistically significant in both groups as regards TC (-16 mg/dL, 95% CI -27, -4, without TDF vs. -28 mg/dL, 95% CI -38, -17, with TDF, p = 0.129 for comparison), TC/HDL ratio (-0.50, 95% CI -0.52, -0.17, and − 0.43, 95% CI -0.72, -0.13, respectively, p = 0.744) and LDL-C (-16 mg/dL, 95% CI -27, -6, and − 24 mg/dL, 95% CI -32, -13, respectively, p = 0.391 for comparison). HDL-C and triglycerides did not significantly vary in strata of current TDF use. Thus, our findings revealed that, using a multivariate model including the age at study entry, the initial level of the variable, the current regimen, the previous regimen, and the concurrent use of lipid lowering drugs, both people who initiated TDF and those who did not, showed similar decline in TC and LDL, and a better lipid profile, suggesting an independent role of DOR.

In the DOR3 group, out of 34 PLWH not on a TAF-including current regimen, 21 took TAF in the previous one (pre-TAF) and 13 did not (no-TAF). We compared them, using the multivariate model previously described. In the current regimen, the proportion of people taking TDF was similar (66.7% and 61.5% respectively). Comparing them, we found that TC declined in both groups (pre-TAF − 22, 95% CI -34, -9, vs. no-TAF − 33 mg/dL, 95% CI -48, -17; p = 0.276), as well as LDL-C (-27 mg/dL, 95% CI -37, -16, vs. -30 mg/dL, 95% CI -42, -16 respectively; p = 0.728). From a statistical point of view, TC/HDL ratio decline was significant in the pre-TAF group (-0.70, 95% CI -1.10, -0.31) and not significant in the no-TAF group (-0.34, 95% CI -0.82, .+0.15), but the two groups were not different (p = 0.238).

In the main analysis (Table 1), HDL-C and triglycerides changes from baseline were not statistically significant and were similar between groups. As regards hepatic safety, in subjects with normal baseline ALT (≤ 40 UI/dL), no groups showed a significant modification in ALT. In those with altered baseline ALT levels, a decline from baseline was observed in DOR3 (-26 UI/L, 95% CI -42, -9, p = 0.005), but no significant difference emerged among groups.

**Table 1:** Baseline values and 6-month follow-up modifications (DOR1: on doravirine switched from rilpivirine-based regimens; DOR2: on doravirine, switching from other NNRTI-containing regimens, DOR3: on doravirine, switching from INSTI-containing regimens)

Variable	DOR1 n = 35	DOR2 n = 32	DOR3 n = 40	P*
**Current regimen**, n (%) 3TC/TDF/DOR Dual with INSTI FTC/TAF/DOR	31 (88.6%) 3 (8.6%) 1 (2.9%)	17 (53.1%) 7 (21.9%) 8 (25.0%)	22 (55.0%) 12 (30.0%) 6 (15.0%)	0.006
**TAF in the previous regimen**, n (%)	5 (14.3%)	6 (18.8%)	26 (65.0%)	< 0.0001
**Blood lipids**				
Total cholesterol, mg/dL				
Baseline, mean ± SD	181 ± 40	203 ± 44	199 ± 46	0.079
*T1-T0, mean (95% CI)*	*-6 (-18, +6)*	***-15 (-26, -5)***	***-23 (-32, -13)***	*0.053*
HDL-cholesterol, mg/dL				
Baseline, mean ± SD	46 ± 13	55 ± 14	49 ± 16	0.020
*T1-T0, mean (95% CI)*	*3 (-1, +8)*	*-2 (-6, +2)*	*-0 (-3, +3)*	*0.059*
Total cholesterol /HDL-cholesterol				
Baseline, median (IQR)	4.0 (3.6–4.7)	3.6 (3.1–4.5)	4.1 (3.4–4.9)	0.339
*T1-T0, mean (95% CI)*	*-0.23 (-0.61,+ 0.14)*	*-0.19 (-0.54, +0.16)*	***-0.45 (-0.75, -0.15)***	*0.435*
LDL-cholesterol, mg/dL				
Baseline, mean ± SD	110 ± 34	119 ± 37	120 ± 42	0.530
*T1-T0, mean (95% CI)*	*-8 (-18, +3)*	***-12 (-22, -3)***	***-22 (-31, -14)***	*0.055*
Triglycerides, mg/dL				
Baseline, median (IQR)	121 (75–145)	107 (89–152)	120 (99–200)	0.156
*T1-T0, mean (95% CI)*	*-9 (-38, +18)*	*-19 (-43, +6)*	*-8 (-30, +14)*	*0.791*
**Liver enzymes**				
ALT ≤ 40 UI/L at T0, n (%)	29 (82.9%)	27 (84.4%)	30 (75.0%)	0.550
Baseline, median (IQR)	21 (19–27)	19 (14–26)	19 (15–26)	0.586
*T1-T0, mean (95% CI)*	*-2 (-7, +2)*	*1 (-3, +6)*	*2 (-1, +6)*	*0.211*
ALT > 40 UI/L at T0, n (%)	6 (17.1%)	5 (15.6%)	10 (25.0%)	0.550
Baseline, median (IQR)	50 (45–63)	49 (42–74)	80 (51–91)	0.260
*T1-T0, mean (95% CI)*	*-18 (-44, +7)*	*10 (-16, +37)*	***-26 (-42, -9)***	*0.084*

3TC: lamivudine; ALT: alanine aminotransferase; CI: confidence interval; DOR: doravirine; FTC: emtricitabine; HDL: high density lipoprotein; INSTI: integrase strand inhibitor; IQR: interquartile range; LDL: low density lipoprotein; TAF: tenofovir alafenamide

**Bold:** change from baseline p < 0.05.

* chi-square test, Mann-Whitney test, or analysis of variance, as appropriate. Changes from baseline were compared among groups using a multivariable linear model including the age at study entry, the initial level of the variable, the current regimen, the concurrent use of lipid lowering drugs and TAF in the previous regimen.

The median time of observation was 12 months (IQR 6–17). Overall, 7 (6.5%) people interrupted the DOR treatment during the first year of observation, 6 because of adverse events (AE) (3 muscle-skeletal, 2 central nervous system, 1 gastrointestinal), and 1 because of virological failure (switched from an RPV-based regimen).

## Discussion

In a real-life context of ART-experienced PLWH, who switched to DOR-containing regimens, we found a generalized improvement of lipid profile, more marked in those who switched from INSTI and NNRTIs other than RPV regimens.

Long-term efficacy and safety of 3TC/TDF/DOR were reported in the context of a trial [13], where reductions in fasting lipids were observed at 24 weeks after the switch and maintained through week 144. Our results confirmed that a better lipid profile was achieved during DOR-based treatment, even accounting for TDF use in the regimen. Moreover, limiting the analysis to those not on TDF in the previous regimen, we observed that TC and LDL-C declined both in people with and without TDF in the current regimen, suggesting an independent effect of DOR in the improvement of lipid profile.

It is well known that TDF could have an independent lipid-lowering effect determining significant decreases in fasting total cholesterol and low-density lipoprotein [14–16]. Improvement in lipid profiles has also been described with TDF compared to other agents in clinical trials [17, 18] and observational studies [19]. Such improvements were also observed in other populations such as HIV-negative men on pre-exposure prophylaxis (PrEP) with TDF/FTC [20] and individuals with chronic hepatitis B treated with TDF [21]. In our sample, we found that, although a more marked decline was observed in PLWH on TDF-including regimen, lipid profile also improved in PLWH on INSTI/DOR and FTC/TAF/DOR, supporting an independent role of DOR. Lastly, in the DOR3 group, we observed that PLWH who withdraw TAF showed a notable improvement in the lipid profile, but even those on a previous regimen without TAF showed a decline in TC and LDL-C, irrespective of TDF presence in the current regimen. This also strengthen the suggestion of an independent effect of DOR on the lipid profile.

We used serum ALT concentration to evaluate the hepatic safety, because PLWH routine care in the participating centers usually only includes this liver function test. However, the serum ALT level remains a very sensitive measure of hepatocellular injury, although not specific, as the degree of elevation cannot determine the exact cause of the damage [22, 23]. In a preliminary analysis of a sample unselected for previous regimen and viral load, we found that ALT showed a slight decrease in PLWH with a baseline ALT level > 40 UI/L [24]. Interestingly, we can confirm that in individuals with elevated baseline levels, ALT declined in those from RPV-containing and INSTI- containing regimens. The latter finding, although statistically significant, derived from a too-small sample (10 study participants) to be significant and needs to be cautiously interpreted and possibly confirmed in larger samples.

This study has some limitations. First, the Infectious Diseases Clinics involved in the SCOLTA study are not formally representative of the Italian Clinics (i.e., at the national level), because they were not randomly selected but participated in our observational study on a volunteer basis. Second, the study participants were not fully representative of all PLWH followed in the Infectious Diseases Clinics participating into the SCOLTA study, but only of those in need of initiating a new ART drug in the considered periods. Despite these limits, our study has the strength to describe a real-life cohort of PLWH on DOR-based regimens, followed up prospectively in multiple centers across Italy, in a research network specifically designed to and with expertise in monitoring the safety of recently marketed antiretroviral drugs.

In conclusion, people on doravirine treatment showed different trends in blood lipids according to the previous regimen they were on. In PLWH switching from RPV, no modification was seen, whereas in those switching from other NNRTIs and from INSTI-including regimens, we observed an overall improvement in lipid profile. This was seemingly independent of the “statin effect” of TDF, being variations similar by TDF use. The ALT reduction in PLWH with altered baseline level needs to be considered with caution and replicated in larger samples.

## Data Availability

Data are available from the corresponding author on reasonable request.

## References

[1] Molina JM, Squires K, Sax PE (2018). Doravirine versus ritonavir-boosted darunavir in antiretroviral-naive adults with HIV-1 (DRIVE-FORWARD): 48-week results of a randomised, double-blind, phase 3, non-inferiority trial. Lancet HIV.

[2] Orkin C, Squires KE, Molina JM (2019). Doravirine/Lamivudine/Tenofovir Disoproxil Fumarate is non-inferior to Efavirenz/Emtricitabine/Tenofovir Disoproxil Fumarate in treatment-naive adults with human immunodeficiency Virus-1 infection: Week 48 results of the DRIVE-AHEAD trial. Clin Infect Dis.

[3] Garcia JM, Dong Y, Richardson P (2023). Effect of HIV and antiretroviral therapy use on body weight changes in a cohort of U.S. veterans living with and without HIV. HIV Med.

[4] Cozzi-Lepri A, Petres L, Pelchen-Matthews (2022). Observational cohort study of rilpivirine (RPV) utilization in Europe. AIDS Res Ther.

[5] Orkin C, Elion R, Thompson M (2021). Changes in weight and BMI with first line doravirine-based therapy. AIDS.

[6] Benedicto AM, Fuster-Martínez I, Tosca J, Esplugues JV, Blas-García A, Apostolova N (2021). NNRTI and liver damage: evidence of their Association and the Mechanisms involved. Cells.

[7] Otto AO, Rivera CG, Zeuli JD, Temesgen Z (2021). Hepatotoxicity of contemporary antiretroviral drugs: a review and evaluation of published Clinical Data. Cells.

[8] Bagella P, De Socio GVL, Ricci E (2018). Durability, safety, and efficacy of rilpivirine in clinical practice: results from the SCOLTA project. Inf Drug Resist.

[9] Casado J, Mena A, Bañón S (2016). Liver toxicity and risk of discontinuation in HIV/hepatitis C virus-coinfected patients receiving an etravirine-containing antiretroviral regimen: influence of liver fibrosis. HIV Med.

[10] Neukam K, Espinosa M, Collado A (2016). Hepatic safety of Rilpivirine/Emtricitabine/Tenofovir Disoproxil Fumarate fixed-dose single-tablet regimen in HIV-Infected patients with active Hepatitis C virus infection: the hEPAtic study. PLoS ONE.

[11] LiverTox. Clinical and research information on drug-induced liver injury. Doravirine. Last update April 2019. Last accessed on February 22, 2023 at: https://www.ncbi.nlm.nih.gov/books/NBK548267/?report=reader

[12] Bonfanti P, Martinelli C, Ricci E, CISAI Group (Italian Coordinators for the Study of Allergies HIV Infection) (2005). An italian approach to post marketing monitoring: preliminary results from the SCOLTA (Surveillance Cohort Long-Term Toxicity Antiretrovirals) project on the safety of lopinavir/ritonavir. J Acquir Immune Defic Syndr.

[13] Kumar P, Johnson M, Molina J-M, Rizzardini G, Cahn P, Bickel M (2021). Brief report: switching to DOR/3TC/TDF maintains HIV-1 virologic suppression through Week 144 in the DRIVE-SHIFT trial. JAIDS J Acquir Immune Defic Syndr.

[14] Tungsiripat M, Kitch D, Glesby MJ (2010). A pilot study to determine the impact on dyslipidemia of adding tenofovir to stable background antiretroviral therapy: ACTG 5206. AIDS.

[15] Randell PA, Jackson AG, Zhong L (2010). The effect of tenofovir disoproxil fumarate on whole-body insulin sensitivity, lipids and adipokines in healthy volunteers. Antivir Ther.

[16] Santos JR, Saumoy M, Curran A (2015). The lipid-lowering effect of tenofovir/emtricitabine: a randomized, crossover, double-blind placebo-controlled trial. Clin Infect Dis.

[17] Gallant JE, Staszewski S, Pozniak AL (2004). Efficacy and safety of tenofovir df vs stavudine in combination therapy in antiretroviral-naive patients: a 3-year randomized trial. JAMA.

[18] Behrens G, Maserati R, Rieger A (2012). Switching to tenofovir/emtricitabine from abacavir/lamivudine in HIV-infected adults with raised cholesterol: effect on lipid profiles. Antivir Ther.

[19] Crane HM, Grunfeld C, Willig JH (2011). Impact of NRTIs on lipid levels among a large HIV-infected cohort initiating antiretroviral therapy in clinical care. AIDS.

[20] Glidden DV, Mulligan K, McMahan V (2018). Metabolic effects of preexposure prophylaxis with coformulated tenofovir disoproxil fumarate and emtricitabine. Clin Infect Dis.

[21] Shaheen AA, AlMattooq M, Yazdanfar S (2017). Tenofovir disoproxil fumarate significantly decreases serum lipoprotein levels compared with entecavir nucleos(t)ide analogue therapy in chronic hepatitis B carriers. Aliment Pharmacol Ther.

[22] Contreras D, González-Rocha A, Clark P, Barquera S, Denova-Gutiérrez E (2023). Diagnostic accuracy of blood biomarkers and non-invasive scores for the diagnosis of NAFLD and NASH: systematic review and meta-analysis. Ann Hepatol.

[23] Moriles KE, Azer SA. Alanine Amino Transferase. In: StatPearls [Internet]. Treasure Island (FL): StatPearls Publishing; 2022 Jan. 2022 Nov 2. Last accessed on February 22, 2023 at: https://www.ncbi.nlm.nih.gov/books/NBK559278/

[24] CISAI Group (Gruppo Coordinamento Italiano Studio Allegrie e Infezione da HIV) (2022). Lipid and transaminase elevation in antiretroviral treatment experienced people living with HIV, initiating a doravirine-based regimen. JHA.

